# Comparison of Microvascular Flow Imaging With Other Doppler Methods in Ultrasonography for Assessment of Parenchymal Perfusion in Undescended Testis in Young Children

**DOI:** 10.7759/cureus.111004

**Published:** 2026-06-16

**Authors:** Suresh Phatak, Akanksha Kalwaghe, Kajal Mitra, Prashant Onkar, Sagar Gund

**Affiliations:** 1 Radiodiagnosis, N. K. P. Salve Institute of Medical Sciences & Research Centre and Lata Mangeshkar Hospital, Nagpur, IND

**Keywords:** color doppler, cryptorchidism, intratesticular blood flow, microvascular imaging, pediatric ultrasonography

## Abstract

Background: Cryptorchidism is a common congenital anomaly in male children, where accurate assessment of testicular vascularity is important for imaging evaluation and characterization of intratesticular blood flow. Conventional Doppler techniques have limitations in detecting low-velocity microvascular flow, particularly in small pediatric testes. These limitations may affect the ability to adequately visualize subtle vascular signals using standard ultrasound techniques.

Methods: This cross-sectional study was conducted at a tertiary care center over six months and included 40 children aged less than five years with clinically diagnosed undescended testis (UDT). Ultrasonography was performed using a high-frequency (2-14 MHz) linear probe of Samsung V7 (Samsung Medison Co., South Korea). Each patient underwent evaluation with Color Doppler (CD), Power Doppler (PD), and Microvascular Imaging (MVI). Doppler settings were optimized (CD/PD pulse repetition frequency (PRF): 520-1000 Hz, MVI PRF: 210 Hz). Intratesticular vascularity was assessed and graded for each modality. Statistical correlation was done using Wilcoxon signed-rank test and McNemar test.

Results: CD detected intratesticular flow in 55% of cases, while PD detected flow in 70%. Both color MVI (cMVI) and monochrome MVI (mMVI) demonstrated significantly higher detection rates (95%). Comparison showed statistically significant superiority of MVI over CD. Although cMVI and mMVI demonstrated similar flow detection rates, mMVI showed a higher proportion of Grade 3 vascularity, indicating superior visualization of intratesticular microvascular flow.

Conclusion: In this prospective study, MVI, particularly mMVI, demonstrated significantly higher intratesticular vascularity grades than conventional CD and PD ultrasonography. mMVI consistently yielded the highest perfusion scores across different anatomical locations of UDT, indicating enhanced visualization and grading of intratesticular microvascular flow. These findings suggest that MVI may serve as a valuable adjunct to conventional ultrasonography in the assessment of testicular perfusion in children with UDT.

## Introduction

Cryptorchidism, or undescended testis (UDT), is one of the most common congenital anomalies in male children, affecting 1-4% of full-term and up to 30% of preterm neonates [1,2]. It refers to incomplete descent of one or both testes into the scrotum and may involve abdominal, inguinal, or high scrotal locations [3,4]. Delayed diagnosis and treatment may result in infertility, hormonal dysfunction, and increased risk of malignancy [5]. Impaired vascular supply and altered temperature regulation may further contribute to progressive testicular damage [6].

Ultrasonography is a non-invasive, widely available, and radiation-free imaging modality that may serve as an adjunctive tool for assessing testicular characteristics and vascularity in selected clinical scenarios, despite its limited role in routine decision-making as per current American Urological Association (AUA) guidelines. High-frequency linear transducers facilitate evaluation of testicular location, size, echotexture, and intratesticular blood flow [7,8].

Color Doppler (CD) and Power Doppler (PD) are commonly used for vascular assessment. While PD is more sensitive to low-velocity flow than CD, both techniques have limited ability to detect slow microvascular flow in small pediatric testes [9-11]. Recent advances have introduced Microvascular Imaging (MVI), also known as Superb Microvascular Imaging (SMI), which improves visualization of low-velocity blood flow without contrast agents [12,13]. MVI uses advanced clutter suppression algorithms to differentiate true vascular signals from motion artifacts and is available as color MVI (cMVI) and monochrome MVI (mMVI), the latter providing better depiction of vascular architecture [14].

Previous studies have demonstrated higher sensitivity of MVI, particularly mMVI, in detecting subtle vascular flow in UDT [15]. The primary objective is detection and grading of intratesticular vascularity and the secondary objectives is comparing the proportion of higher vascularity grades across modalities. 

This work was previously presented as a paper at the Indian Radiological and Imaging Association (IRIA) conference on January 29, 2026. The presentation included the complete dataset and full analysis of the study. However, no abstract or conference proceedings related to this presentation have been published.

## Materials and methods

Study design and setting

This hospital-based cross-sectional study was conducted in the Department of Radiodiagnosis at N.K.P. Salve Institute of Medical Sciences & Research Centre and Lata Mangeshkar Hospital (NKPSIMS & RC and LMH), Nagpur, India over a period of six months, from March 2025 to August 2025. The study was approved by the Institutional Ethics Committee of NKPSIMS & RC and LMH (Approval No: NKPSIMS & RC and LMH/6/2025; Date: January 30, 2025). Informed written consent was obtained from the parents or guardians of all participating children.

Study population

A total of 40 male children aged less than five years old with clinically suspected UDT were included in the study. Clinical examination was performed prior to ultrasonographic evaluation to assess testicular palpability and probable location. Demographic details including age, laterality, and site of UDT were recorded.

Inclusion and exclusion criteria

Children with clinically suspected unilateral or bilateral UDT were included in the study. The unit of analysis in the present study was the testis. A total of 40 testes from 40 different patients were included in the final analysis. Although a few patients presented with bilateral cryptorchidism, only one UDT per patient was included to maintain a single independent observation per participant and to avoid potential clustering bias. Therefore, each observation represented an independent testicular unit, and no patient contributed more than one testis to the statistical analysis. Of the 40 patients included, 35 presented with unilateral and five with bilateral UDT. Normally descended contralateral testes were identified and confirmed in normal scrotal position on grayscale imaging in all patients but were not formally graded for vascularity and were not used as internal controls, as comparative assessment of descended and UDT was beyond the scope of the present study

Patients with prior inguinoscrotal surgery, testicular torsion, scrotal trauma, acute infection affecting vascularity, known testicular neoplasm, or inadequate ultrasonographic visualization were excluded. 

Among the initially screened patients, three were excluded based on predefined exclusion criteria: two had a history of prior inguinoscrotal surgery and one was excluded due to inadequate ultrasonographic visualization. None of the screened patients presented with testicular torsion, scrotal trauma, testicular neoplasm, or acute infection affecting vascular assessment. The remaining 40 patients who fulfilled all inclusion criteria were enrolled and form the final study cohort. All included examinations were performed using a standardized imaging protocol to ensure adequate visualization and consistent microvascular assessment.

Ultrasonographic technique

All Doppler examinations were performed using the Samsung V7 ultrasound system (Samsung Medison Co., Ltd., South Korea) with a high-frequency linear transducer (LA2-14A, 2-14 MHz) with the patient in the supine position in a warm room to minimize cremasteric contraction and motion artifacts. Vascular evaluation was performed using a standardized fixed sequence in all examinations: CD, followed by PD, cMVI, and mMVI. Assessments were performed during real-time scanning.

Imaging depth was adjusted individually for each patient according to testicular size and location, ensuring complete visualization of the testicular parenchyma. MVI acquisitions were performed at a reduced depth compared to CD and PD to further optimize spatial resolution for detection of low-velocity intratesticular flow. B-mode gain was set to 70-100 for CD/PD and 60 for MVI; dynamic range was 90-110 dB and 110-140 dB respectively; frame average was 5-6; and persistence was maintained at 90-92% throughout.

Initial grayscale ultrasonography was performed to identify the location of the testes and evaluate testicular size, echogenicity, and echotexture. The inguinal canal, deep inguinal ring, lower abdomen, and scrotum were systematically examined in all patients.

For CD and PD, pulse repetition frequency (PRF) settings between 520-1000 Hz were used, while MVI evaluation was performed at a PRF of 210 Hz for improved visualization of low-velocity microvascular flow. The color box was positioned to encompass the entire testicular parenchyma, and the focal zone was centered within the color box. Doppler gain was optimized just below the noise threshold for each modality by increasing gain until random noise appeared and then reducing it until noise disappeared. In MVI mode, wall filtering is not available as a separately adjustable parameter; clutter suppression is instead managed through the system's integrated proprietary algorithm, as per the manufacturer's design specifications of the Samsung V7 platform. No extrinsic image processing was applied, and preset values were preserved throughout each examination. Safety indices were maintained within acceptable limits throughout (Soft Tissue Thermal Index (TIs) ≤ 0.5, Bone Thermal Index (TIb) ≤ 0.5, Mechanical Index (MI) = 1.4).

Assessment of vascularity

All ultrasonographic examinations and Doppler vascularity grading were performed prospectively during real-time scanning by a single radiologist with (30 years) of experience in Doppler ultrasonography and pediatric imaging. Vascularity grades (0-3) were assigned at the time of examination using live imaging.

**Figure 1:**
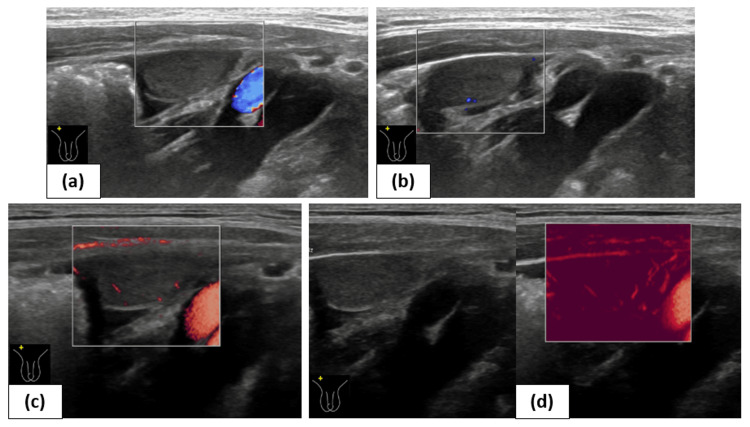
A two-year-old patient with swelling in right inguinal region. (a) CD shows grade 0 flow; (b) PD shows one spot color encoding signal, i.e., Grade 1 parenchymal flow; (c) cMVI shows a linear and two spot color encoding signals, i.e., Grade 2 flow; (d) mMVI shows multiple linear and spot color encoding signals, i.e., Grade 3 flow. CD: Color Doppler; PD: Power Doppler; cMVI: Color Microvascular Imaging; mMVI: Monochrome Microvascular Imaging

**Figure 2:**
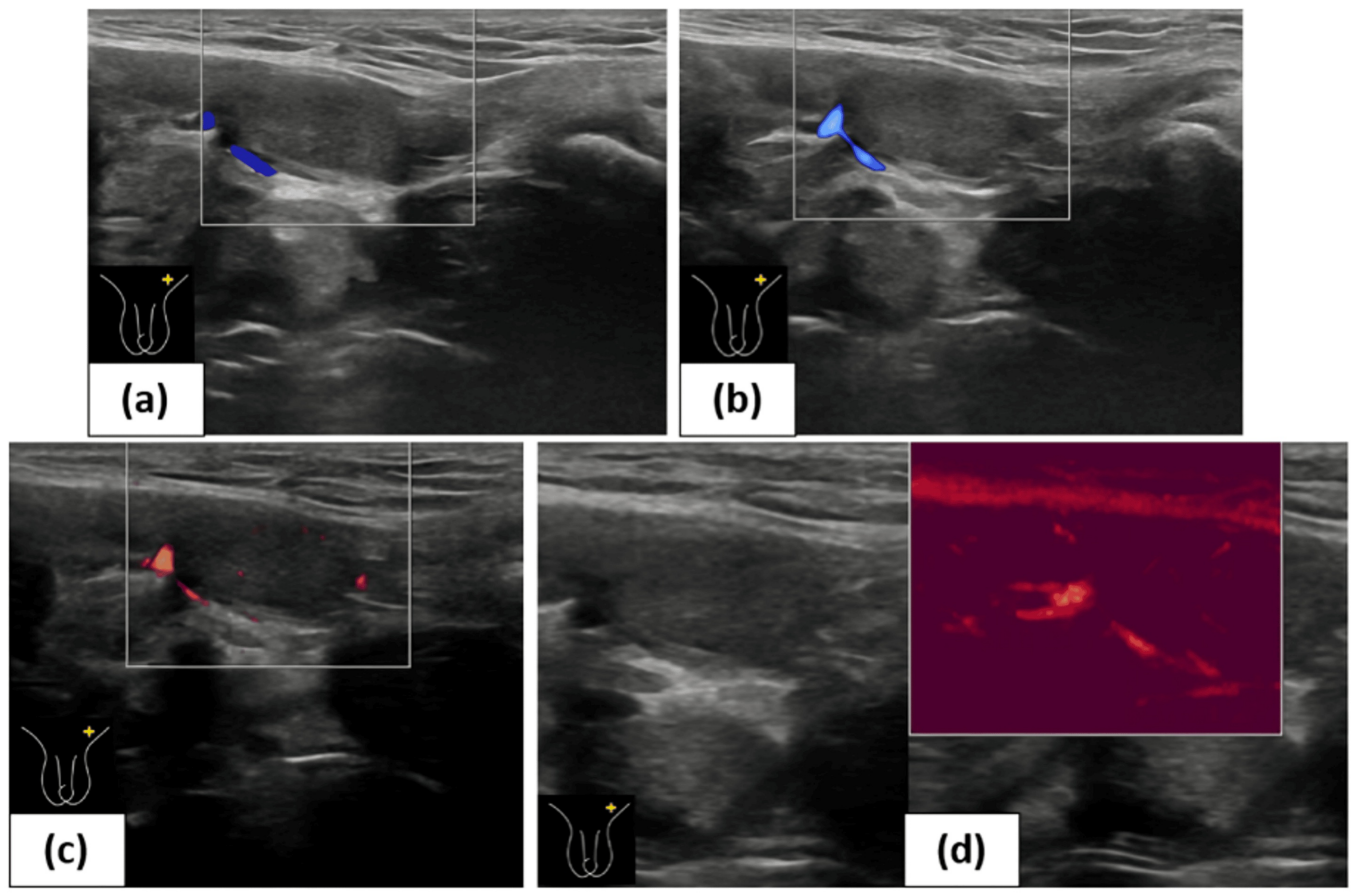
A 18-month-old male patient presented with left inguinal swelling (a) CD shows Grade 0 flow; (b) PD shows Grade 0 parenchymal flow; (c) cMVI shows single spot color encoding signal, i.e., Grade 1 flow; (d) mMVI shows few linear and spot color encoding signals, i.e., Grade 3 flow. CD: Color Doppler; PD: Power Doppler; cMVI: Color Microvascular Imaging; mMVI: Monochrome Microvascular Imaging

Intratesticular vascularity was assessed by documenting the presence of flow and grading vascular signals (Table 1) for each modality (Figures 1, 2). The diagnostic performance of CD, PD, and MVI was compared in detecting microvascular flow.

**Table 1:** Testicular blood flow was categorized into four distinct grades based on Doppler ultrasound findings and this grading system was uniformly applied across all patients regardless of the Doppler technique used Adapted from L. Karaca et al. [16] with permission from original publisher (published under the Creative Commons CC BY-NC-ND 4.0 license.)

Grade	Definition of Intratesticular Vascularity
Grade 0	No vascularity
Grade 1	Presence of one or two spots color encoding
Grade 2	Presence of either a single linear color encoding or several spot color encoding
Grade 3	Presence of more than one linear color encoding

Statistical analysis****

Data were analyzed using IBM Statistical Package for the Social Sciences (SPSS) software version 25 (IBM Corp., USA). Since all four Doppler modalities - CD, PD, cMVI, and mMVI - were applied to the same patients, all comparisons were treated as paired observations. Vascularity parameters were recorded on an ordinal scale (0-3); paired ordinal comparisons across all six modality pairs were therefore performed using the Wilcoxon signed-rank test, which utilizes the full magnitude of ranked differences and avoids loss of information inherent in dichotomization. In addition, binary flow detection (present versus absent, defined as score ≥1) was compared across modalities using the McNemar exact test as a supplementary analysis. Since six pre-specified pairwise comparisons were performed, Bonferroni correction was applied to adjust for multiple comparisons, yielding a corrected significance threshold of p<0.0083 (0.05÷6). All p-values are reported as calculated; statistical significance was determined against the Bonferroni-corrected threshold throughout.

## Results

In the present study, among the age group distribution, 10 cases (25.00%) were less than one year old, 18 cases (45.00%) were one to two year olds, and 12 cases (30.00%) were more than two years old. With respect to site of UDT, eight cases (20.00%) each were located in right high inguinal, right low inguinal, and left high inguinal regions, six cases (15.00%) each in right abdominal and left low inguinal regions, and four cases (10.00%) in left abdominal region; regarding palpability, 24 cases (60.00%) were palpable and 16 cases (40.00%) were non-palpable; on CD, Grade 0 flow was observed in 18 cases (45.00%), Grade 1 in 14 cases (35.00%), and Grade 2 in eight cases (20.00%). On PD, Grade 0 flow was seen in 12 cases (30.00%), Grade 1 seen in 12 cases (30.00%), and Grade 2 was seen in 16 cases (40.00%). On cMVI, Grade 0 flow was noted in two cases (5.00%), Grade 1 was seen in six cases (15.00%), Grade 2 seen in 22 cases (55.00%), and Grade 3 seen in 10 cases (25.00%). On mMVI, Grade 0 flow was seen in two cases (5.00%), Grade 1 was seen in two cases (5.00%), Grade 2 was seen in 12 cases (30.00%), and Grade 3 was seen in 24 cases (60.00%) (Table 2). 

**Table 2:** Distribution of study population according to demographic, clinical characteristics, and Doppler parameters CD: Color Doppler; PD: Power Doppler; cMVI: Color Microvascular Imaging; mMVI: Monochrome Microvascular Imaging

Parameter	Category	n	%
Age group	<1 year	10	25.00%
	1-2 years	18	45.00%
	>2 years	12	30.00%
Site	Right high inguinal	8	20.00%
	Right low inguinal	8	20.00%
	Left high inguinal	8	20.00%
	Right abdominal	6	15.00%
	Left low inguinal	6	15.00%
	Left abdominal	4	10.00%
Palpable	Present	24	60.00%
	Absent	16	40.00%
CD	Grade 0	18	45.00%
	Grade 1	14	35.00%
	Grade 2	8	20.00%
PD	Grade 0	12	30.00%
	Grade 1	12	30.00%
	Grade 2	16	40.00%
cMVI	Grade 0	2	5.00%
	Grade 1	6	15.00%
	Grade 2	22	55.00%
	Grade 3	10	25.00%
mMVI	Grade 0	2	5.00%
	Grade 1	2	5.00%
	Grade 2	12	30.00%
	Grade 3	24	60.00%

Table 3 demonstrates the comparison of different Doppler modalities to detect intratesticular vascular flow. Conventional CD showed the lowest detection rate, while PD demonstrated improved sensitivity for vascular detection. Although cMVI and mMVI demonstrated identical overall flow detection rates (95%), mMVI showed a greater proportion of Grade 3 vascularity (60% vs. 25%), suggesting improved depiction of intratesticular microvascular architecture.

**Table 3:** Comparative flow detection rates across CD, PD, cMVI, and mMVI modalities CD: Color Doppler; PD: Power Doppler; cMVI: Color Microvascular Imaging; mMVI: Monochrome Microvascular Imaging

Modality	Flow detected (Grade ≥1)	Detection Rate
CD	22/40	55%
PD	28/40	70%
cMVI	38/40	95%
mMVI	38/40	95%

Doppler vascularity scores varied according to the anatomical location of the UDT (Table 4). Right low inguinal testes demonstrated the highest vascularity (mMVI = 3.00), followed by left high inguinal and left low inguinal testes. Right high inguinal and left abdominal testes showed moderate vascularity, whereas right abdominal testes exhibited the lowest perfusion (mMVI = 1.67) with absent CD flow. Overall, vascularity tended to decrease with increasing proximal location of the UDT.

**Table 4:** Doppler vascularity parameters of UDT according to site of maldescent Sites are arranged in descending order of mean mMVI score. UDT: Undescended testis; CD: Color Doppler; PD: Power Doppler; cMVI: Color Microvascular Imaging; mMVI: Monochrome Microvascular Imaging; n: Number of cases

Site of UDT	No. of Cases	Mean CD	Mean PD	Mean cMVI	Mean mMVI	Vascularity Pattern
Right low inguinal	8	1.25	1.75	2.5	3	High
Left high inguinal	8	1.38	1.75	2.12	2.88	Moderately high
Left low inguinal	6	0.5	0.67	2.17	2.83	Moderately high
Right high inguinal	8	0.5	0.75	1.75	2	Moderate
Left abdominal	4	0.5	0.5	2	2	Moderate
Right abdominal	6	0	0.67	1.33	1.67	Low-moderate
Total	40					

Wilcoxon signed-rank test was applied to ordinal vascularity scores (0-3), while McNemar test was used for binary vascularity detection (present vs absent) (Table 5). Bonferroni correction for six paired comparisons yielded a significance threshold of p<0.0083. All Wilcoxon comparisons remained significant after correction. McNemar results with p≥0.0083 were considered non-significant. Partial concordance indicates discordant significance between ordinal and binary analyses.

**Table 5:** Wilcoxon signed-rank (ordinal) and McNemar (binary) test results with Bonferroni correction for multiple comparisons (n=40) A p-value of <0.0083 (Bonferroni-corrected threshold; 0.05÷6) was considered statistically significant for all comparisons. CD: Color Doppler; PD: Power Doppler; cMVI: Color Microvascular Imaging; mMVI: Monochrome Microvascular Imaging

Comparison	Wilcoxon p-value	Wilcoxon Result	McNemar p-value	McNemar Result	Interpretive Conclusion
CD vs PD	0.0011	Significant	0.031	Not significant after correction	Partial concordance - PD demonstrates superior ordinal vascularity grading over CD; however binary detection rates are not significantly different after correction
CD vs cMVI	<0.0001	Highly significant	0.001	Highly significant	Concordant - cMVI significantly outperforms CD across both ordinal grading and binary detection
CD vs mMVI	<0.0001	Highly significant	0.001	Highly significant	Concordant - mMVI significantly outperforms CD across both ordinal grading and binary detection
PD vs cMVI	<0.0001	Highly significant	0.002	Significant	Concordant - cMVI significantly outperforms PD across both ordinal grading and binary detection
PD vs mMVI	<0.0001	Highly significant	0.002	Significant	Concordant - mMVI significantly outperforms PD across both ordinal grading and binary detection
cMVI vs mMVI	0.0001	Highly significant	1	Not significant	Partial concordance - mMVI yields significantly higher perfusion grades than cMVI; however binary detection rates are identical, indicating mMVI provides superior quantitative grading without improving case detection

## Discussion

Cryptorchidism is one of the most common congenital anomalies in male children and is associated with infertility, hormonal dysfunction, and increased risk of malignancy if left untreated [1-6]. Assessment of intratesticular vascularity may provide additional information regarding testicular health, as impaired blood flow could be associated with testicular damage and reduced viability. Ultrasonography remains the preferred initial imaging modality for evaluating UDT due to its non-invasive nature, wide availability, and ability to assess testicular morphology and vascularity. Although it is not routinely recommended in the standard evaluation of cryptorchidism, advanced techniques such as MVI may have a role in selected clinical scenarios [7,8]. However, conventional Doppler techniques have limitations in detecting slow microvascular flow in small pediatric testes [9-11]. The present study compared CD, PD, cMVI, and mMVI in evaluating intratesticular vascularity in children with cryptorchidism.

In the present study, most patients belonged to the 1-2-year age group, and the inguinal canal was the most common location of UDT. These findings are consistent with previous studies describing the inguinal region as the most frequent site of testicular arrest during descent [3,4]. A significant proportion of testes were non-palpable, highlighting the importance of imaging for localization and vascular assessment.

Among the evaluated Doppler techniques, CD demonstrated the lowest sensitivity for vascular detection, with absent flow in 45% of cases. PD showed improved vascular detection compared with CD, reflecting its greater sensitivity to low-velocity flow. Similar observations were reported by Martinoli et al., who demonstrated the improved ability of PD to detect weak vascular signals compared with CD [10]. Barth and Shortliffe also reported superior detection of testicular blood flow using PD in pediatric patients [11]. Despite improved performance, PD still failed to identify vascularity in several cases, indicating limitations in detecting very slow microvascular flow.

The most important finding of the present study was the markedly superior performance of MVI techniques, particularly mMVI, in demonstrating intratesticular vascularity. Both cMVI and mMVI detected vascular flow in nearly all cases, with mMVI showing the highest proportion of Grade 3 vascularity. These findings support previous studies demonstrating the superiority of MVI techniques in evaluating low-flow vascular signals [12-14].

Our findings are comparable with those of Ates et al. and Karaca et al., who demonstrated improved detection and visualization of intratesticular microvascular flow using SMI compared with conventional Doppler techniques in UDT and pediatric testes [15,16]. The most important finding of the present study was the markedly superior performance of MVI techniques in demonstrating intratesticular vascularity. While cMVI and mMVI showed similar overall flow detection rates, mMVI demonstrated a higher proportion of Grade 3 vascularity, suggesting superior visualization of microvascular flow patterns.

From a clinical perspective, improved detection of intratesticular vascularity with MVI may provide additional information during ultrasonographic evaluation of cryptorchid testes. Demonstration of preserved vascular signals may complement conventional assessment of testicular morphology and location. However, the present study evaluated flow detection capability only and was not designed to determine the effect of MVI findings on surgical decision-making or clinical outcomes. Further studies incorporating surgical correlation and long-term follow-up are required to establish the clinical role of MVI in cryptorchidism management. Therefore, incorporation of MVI as an adjunctive ultrasonographic technique may improve visualization of intratesticular vascularity and enhance diagnostic confidence in children with cryptorchidism.

Limitations

The present study has certain limitations. The sample size was relatively small and the study was conducted at a single center, which may limit generalizability of the findings. Ultrasonography and Doppler evaluation are operator-dependent techniques and may be influenced by patient movement and cooperation, particularly in young pediatric patients.

Histopathological correlation and long-term clinical follow-up were not available in all cases, limiting definitive validation of imaging findings and assessment of prognostic implications. Evaluation of deeply located abdominal testes may also be technically challenging, which could potentially affect accurate vascular assessment.

In addition, all image grading was performed by a single reader, and interobserver variability was not assessed, which may introduce observer bias. Although SMI demonstrated improved visualization of intratesticular vascularity compared to conventional Doppler techniques, the study does not evaluate whether this improved detection directly translates into changes in clinical management or patient outcomes.

## Conclusions

The present study demonstrates that MVI, including both cMVI and mMVI, detects flow more frequently and at higher grades as compared to conventional Doppler techniques in UDT. While CD showed the lowest sensitivity and PD provided moderate improvement, both techniques were limited in identifying low-velocity microvascular flow. In contrast, MVI techniques exhibited significantly higher flow detection rates than conventional Doppler methods. Although cMVI and mMVI demonstrated similar detection rates, mMVI showed a greater proportion of Grade 3 vascularity, suggesting superior visualization of microvascular perfusion. These findings highlight the limitations of conventional Doppler methods in pediatric testicular evaluation and suggest that MVI may serve as a more sensitive and reliable modality. Accurate assessment of intratesticular vascularity may aid in improving preoperative counseling. Therefore, incorporation of MVI into routine ultrasound evaluation may serve as a more sensitive adjunct in affected children.
